# The Army Medical Service Inquiry

**Published:** 1917-09-01

**Authors:** 


### The Hospital

The Workers' Newspaper of Administrative Medicine and Institutional Life, Administration, National Insurance and Health.

No. 1630, Vol. LXII. SATURDAY, SEPTEMBER 1, 1917. PRICE ONE PENNY

### THE ARMY MEDICAL SERVICE INQUIRY.

For some time it has been evident that the continued demand of the Army for an increasing number of medical officers must sooner or later reach a point at which would be put in peril the medical interests of the civilian population. This point has now been reached, as is indicated by the announcement of the Central Medical War Committee of their inability under existing conditions to provide any more doctors for military service. There would, therefore, at least at first sight, seem no escape from the conclusion that either the soldiers or the civilians must accommodate themselves to a quantitatively inefficient medical service. On the one side the Army authorities say they must have more doctors, on the other the Central Medical War Committee announce the impossibility of supplying them. Thus it would seem we have arrived at a deadlock. It therefore becomes necessary to scrutinise with great care the rival positions. Now one of these positions, namely that of the War Committee, is based upon a minute and exact inquiry—an inquiry which has analysed the possibilities of every practitioner in the country who is engaged in civil practice. Time and knowledge and care have been applied to the investigation, and the conclusion has been accepted as beyond challenge. No such claim has been made or can be made on the part of the Army. All that we have is the general assurance that more doctors are needed. To accept this assurance without investigation is in the circumstances impossible, and this the more so that it involves two very serious consequences. The first of these is a further reduction of the medical provision necessary for the welfare of the civil population; and the second, the application of new compulsory powers to the civilian members of the profession. Public opinion, as we have said on more than one occasion, would not, we are convinced, support either of these proposals unless the necessity for them was made clearly evident. And such necessity could only become evident when the work of the doctors engaged in military duties has been examined in the thorough and detailed fashion which has already been applied to the doctors occupied in civil practice.

This position, at least in its broad outlines, has now been accepted by the Government, and the Committee of Inquiry which has been appointed is to carry out its investigations first of all in France, and afterwards in Great Britain. Let us at the outset say that the appointment of the Committee does not cast any reflection on the military medical authorities. These have had to work under great pressure, and their responsibility is a very heavy one. Further, it has to be remembered that the situations created by the war are largely without precedent. Both medical officers and combatant officers had no substantial body of experience to equip themselves in detail for their present tasks. They have had to meet entirely novel and unexpected emergencies, and this, too, while engaged in the discharge of a laborious daily routine. There is no suggestion of defect either in their goodwill or in their efficiency in the proposal that a survey of the position by fresh minds may possibly detect methods of organisation which will produce some economy of effort. Still more important is the fact that to grant their demands means harm to members of the civilian community. It is their immediate duty to provide for the soldiers, and this stands for them as the first claim. But obviously the new situation which has arisen necessitates a wider view, and hence, while listening with great respect to their claim, we must refer a judgment on it to a tribunal which has access to all the evidence. If no other way can be discovered, we agree that the civilians must suffer rather than the soldiers. But before accepting any responsibility for such suffering the need for it must be made manifest in the sight of all men. Of the good faith of the military medical authorities no one has any doubt. The question at issue is whether their necessities may not perhaps be met by methods and adjustments which will avoid the further conscription of the medical profession and the sacrifice of the health interests of civilians.

On the composition of the Committee of Inquiry we have no word to say. The Committee has been called into existence for a very practical purpose and it will be judged by its methods and by its achievements. Distinction in other fields, personal or professional, is a subordinate matter, and no one will be concerned about it either one way or the other. The first demand clearly is to discover the facts, for it is quite certain that no mere general statements, however comfortable the phrasing, will satisfy either the profession or the public. In this connection we urge the importance of taking the evidence of men who have served for a year or longer as temporary officers in the R.A.M.C. These men can give personal experiences, some of them from work actually at the fighting front, others from service in unexposed situations. Further, from such sources can be gained valuable estimates of the amount of work which in existing circumstances can reasonably be expected from a practitioner. These men know from personal trial what to-day is the daily service both of the civil practitioner and of the military medical officer. They are free to speak and their evidence is essential. There is, of course no real conflict between the civilians and the soldiers. But the doctors exist only in limited numbers and it is a claim of high policy as well as of humanity that they shall be distributed in the most effective manner possible The Committee will have this end in view, and the more clearly they keep it in view the more likely are they to justify their appointment.

